# ﻿The ants of the genus *Rhopalothrix* Mayr, 1870 (Hymenoptera, Formicidae, Myrmicinae) in Colombia

**DOI:** 10.3897/zookeys.1191.110418

**Published:** 2024-02-13

**Authors:** Roberto J. Guerrero, Andrés F. Grajales-Andica, Fernando Fernández, María C. Tocora, Gianpiero Fiorentino, Delly R. García

**Affiliations:** 1 Universidad del Magdalena, Facultad de Ciencias Básicas, Programa de Biología, Santa Marta, Magdalena, Colombia Universidad del Magdalena Santa Marta Colombia; 2 Departamento de Ecología y Recursos Naturales, Centro Universitario de la Costa Sur, Universidad de Guadalajara, Autlán de Navarro, Mexico Universidad de Guadalajara Autlán de Navarro Mexico; 3 Instituto de Ciencias Naturales, Universidad Nacional de Colombia, Bogotá, D.C., Colombia Universidad Nacional de Colombia Bogotá Colombia; 4 Department of Ecology and Evolutionary Biology, University of Toronto, Toronto, ON M5S 3B2, Canada University of Toronto Toronto Canada; 5 New Jersey Institute of Technology, New Jersey, USA New Jersey Institute of Technology New Jersey United States of America; 6 Universidad del Quindío, Armenia, Quindío, Colombia Universidad del Quindío Armenia Colombia

**Keywords:** *Basiceros* genus group, identification key, *isthmica* clade, new species, South America, taxonomy

## Abstract

The ants of the genus *Rhopalothrix* are diverse in the Neotropical region, with 14 of the 16 described species. Based on museum material and recent fieldwork, *Rhopalothrix* ants in Colombia were reviewed. Morphological analysis of the workers allowed delimitation of six species, including two new species, *Rhopalothrixmandibularis* Guerrero & Grajales, **sp. nov.** and *Rhopalothrixmariaemirae* Tocora, Fiorentino & Fernández, **sp. nov.** A new combination *Rhopalothrixamati***comb. nov.** is proposed for *Eurhopalothrixamati*. A worker-based taxonomic key, high-definition images of the workers, and a distribution map of all *Rhopalothrix* species present in Colombia are provided.

## ﻿Introduction

Ants are a dominant and ecologically key component of the highly diverse fauna of mostly exceedingly small arthropods that live in the litter layer that accumulates on the forest floor. Habitat type, as well as leaf-litter quality and heterogeneity, can influence the ant community (Silva et al. 2011), allowing some genera of ants to become more conspicuous (e.g., *Pheidole* Westwood, 1839 or *Strumigenys* Smith, 1860), while others are cryptic and poorly represented, such as *Rhopalothrix* Mayr, 1870.

The *Basiceros* genus group contains the genera *Basiceros* Schulz, 1906, *Eurhopalothrix* Brown & Kempf, 1961, *Octostruma* Forel, 1912, *Protalaridris* Brown, 1980, *Rhopalothrix*, and *Talaridris* Weber, 1941. The ants of the genus *Rhopalothrix* are small and with a distinctive combination of features. The worker mandible is an arched shaft with an apical fork; most other members of the *Basiceros* genus group have triangular mandibles. The genus *Protalaridris* has elongate mandibles, similar to *Rhopalothrix*, but can be distinguished by their antennae with 9 segments, instead of 7 in *Rhopalothrix*. *Rhopalothrix* workers also have squamiform setae varying in number and size on the head, mesosoma or gaster.

Currently, sixteen species are recognized within *Rhopalothrix* (Bolton 2023), but at least a dozen species await description (AntWeb 2023). The genus *Rhopalothrix* is distributed in Australia (1 species), New Guinea (1 species) and the Neotropical region (14 species) (Longino and Boudinot 2013). Longino and Boudinot (2013) studied *Rhopalothrix*, mainly the Mesoamerican forest fauna. These authors proposed the *Rhopalothrixisthmica* clade, defined by two synapomorphies: absence of squamiform setae on the face and development of shallow arcuate grooves and ridges on the face. This clade contains 13 of the 14 described Neotropical species. Several additional Neotropical species of uncertain phylogenetic position are now known, with characters that place them outside the *isthmica* clade (see images on AntWeb 2023).

We describe two new species, one that fits in the *isthmica* clade and another with scale-like setae on the face, similar to those present in *Rhopalothrixciliata* Mayr, 1870. We also propose a new combination for one species previously described in the genus *Eurhopalothrix*. We provide a key to the six Colombian species, new occurrence records, and results on the distribution of the species in the country.

## ﻿Materials and methods

### ﻿Specimen processing

**Figure 1.**
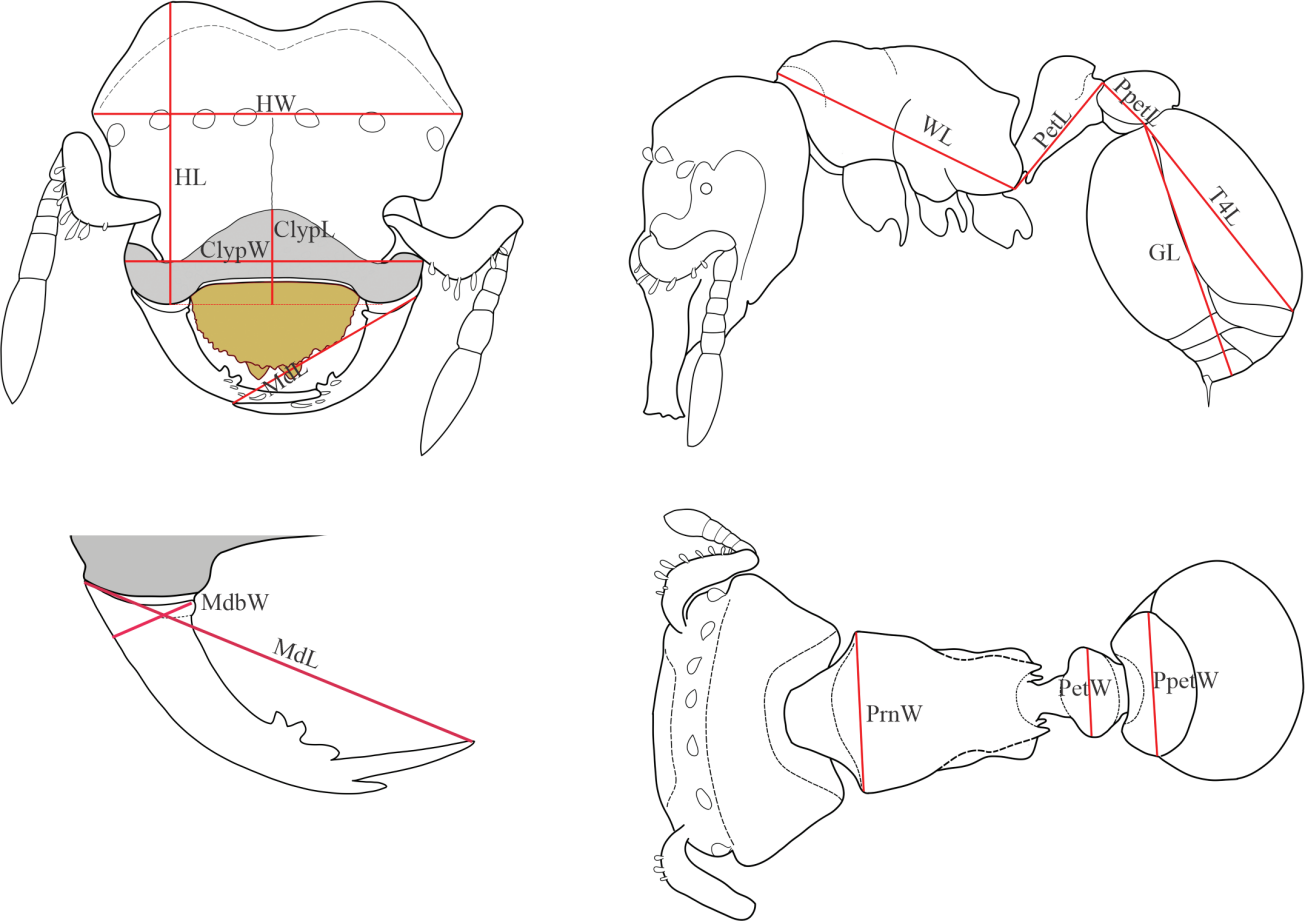
Measurements recorded in the habitus of *Rhopalothrix* worker. Definitions of the acronyms are described in Material and methods.

We used the worker-based key to species of *Rhopalothrix* proposed by Longino and Boudinot (2013) for identification of the studied specimens. To integrate our data into a review of the genus *Rhopalothrix* from the Brazilian Atlantic and Amazonian rainforest (J. Chaul, personal communication) and better characterize the species described here, we implemented several complementary measurements (for definitions see below; Fig. 1) to the one used by Longino and Boudinot (2013). The latter only used the maximum width of the head capsule in full-face view (HW) as a surrogate measure of ant size. Although HW is useful for the separation of known species of *Rhopalothrix*, the morphological diversity within the genus requires the exploration of other measures to support the delimitation of new taxa.

Specimens were observed using a Nikon SMZ 745 stereomicroscope. Measurements were made with a dual-axis micrometer stage with output in increments of 0.001 mm. However, variation in specimen orientation, alignment of crosshairs with edges of structures, and interpretation of structure boundaries resulted in measurement accuracy to the nearest 0.01 mm. All measurements (Fig. 1) are presented in mm:

**ClyL** in full-face view, maximum width of the clypeal plate including the lateral expansions above the insertion of the mandibles.

**ClyW** in full-face view, maximum length of the clypeal plate from the most anteroclypeal projection to the most posterior clypeal margin.

**GL** in lateral view, the straight-line length of the gaster measured from the most anterior margin of the first tergite to the posterior margin of the fourth tergite.

**HL** in full-face view, maximum length of the head measured from the most anterior projection of the clypeus to the most posterior projection of the cephalic capsule.

**MdL** in full-face view, the straight-line length of the mandible from the basalmost mandibular external margin to the apex of the subapical tooth.

**MdbW** in full-face view, shortest diagonal line connecting the most basal point of the masticatory margin with the mandibular external margin.

**PetL** in profile view, the distance from the inflection point marking the juncture of the cylindrical posterior portion of the segment to the anterior inflection point where the petiole is obscured by the posteroventral lobes of the propodeum.

**PpetL** in lateral view, the distance from anterior to posterior inflections of postpetiole node.

**PetW** maximum width of the petiolar node in dorsal view.

**PpetW** maximum width of the postpetiolar node in dorsal view.

**PrnW** maximum width of the pronotum in dorsal view.

**T4L** in lateral view, length of the fourth abdominal tergite (= first gastral tergite) measured with the anterior and posterior margins in the same plane.

**WL** the diagonal length of the mesosoma in profile from the point at which the pronotum meets the cervical shield to the posterior basal angle of the metapleuron.

The taxonomic key provided here includes the relationship between labral width (LabW) and labral length (LabL) (see also couplet 4 in Longino and Boudinot (2013)). In full-face view, we measured the width (LabW) and the length of the labrum (LabL) in those specimens corresponding to *Rhopalothrixisthmica* (Weber, 1941), *R.mandibularis* sp. nov. and *R.weberi* Brown & Kempf, 1960. The values are expressed as a percentage, (LabW/LabL)*100. When preparing specimens of both species it is recommended to carefully open the mandibles so that the labrum is completely exposed.

High-resolution images of *Rhopalothrixciliata* Mayr, 1870 lectotype (CASENT0915695) and *Rhopalothrixmariaemirae* sp. nov. (= *Rhopalothrix* jtl021: ANTWEB1038216, UFV-LABECOL-001953 and USNMENT01127994) workers were downloaded from http://www.antweb.org. For each image, we record all the measurements indicated above using Image J software (NIH, Bethesda, MD, USA). For the *R.ciliata* lectotype, only those measurements of the head, mandible, and mesosomal and petiolar/postpetiolar dorsum were recorded; those measurements taken in lateral view could not be recorded due to the position of the specimen on the pin.

The global distribution of *Rhopalothrix* was obtained from AntMaps (Janicki et al. 2016). The distribution map for all species of *Rhopalothrix* in Colombia was made with R software (R Core Team 2020), using information from specimen collection labels. The shapefiles were extracted from the *rnaturalearth library World Map Data* from Natural Earth v. 0.3.3 (Massicotte 2024). The digital elevation model was developed using the geodata library. The final map presented here was obtained using the graphical tool “ggplot2”.

For comparative purposes, type, and non-type specimens of different *Rhopalothrix* species were studied from high-quality images downloaded from www.antweb.org (AntWeb 2023); unique specimen numbers are provided in all cases.

### ﻿Specimen drawing, imaging, Micro-CT scanning and 3D-reconstruction

Drawings of the general habitus of *Rhopalothrix* with measurements, and the mandibular apical fork of each species recorded here were created using Adobe Sketchbook v. 9.0.

Color montage images of the species were created using an Auto-Montage Leica M205A and the images were combined using the program LAS v. 4.6. The images were edited (Corel Photo–Paint X3 v. 13.0) to enhance brightness and contrast details. Finally, all figures were arranged using CorelDRAW Graphics Suite X3.

Micro-CT scans of a specimen of *Rhopalothrixmariaemirae* sp. nov. were generated with a Zeiss Xradia 510 Versa 3D X-ray microscope operated with the Zeiss Scout-and-Scan Control System software (v. 14.0.14829.38124). The scan was carried out at the Okinawa Institute of Science and Technology Graduate University, Japan. Scans were conducted with a 40 kV (75 μA) / 3 W beam using the 4x magnification objective. The scan was performed at an exposure time of 25 s and a voxel size of 0.645545 μm.

### ﻿Repositories

We examined specimens deposited in the following collections:

**CBUMAG**Colecciones Biológicas de la Universidad del Magdalena, Santa Marta, Magdalena, Colombia.

**CELC**Coleção Entomológica do Laboratório de Sistemática e Biologia de Coleoptera, Universidade Federal de Viçosa, Viçosa, Brazil.

**CPDC**Centro de Pesquisas del Cacao, Comissão do Plano de Lavoura, Itabuna, Bahia, Brazil.

**CTNI** Colección Taxonómica Nacional de Insectos Luis María Murillo, Corporación Colombiana de Investigación Agropecuaria – AGROSAVIA, Tibaitatá, Mosquera, Cundinamarca, Colombia.

**DZUP** Coleção Entomológica Padre Jesus Santiago Moure, Universidade Federal do Paraná, Curitiba, Brazil

**JTLC**John T. Longino, personal collection, University of Utah, Salt Lake City, UT, USA.

**IAvH** Instituto de investigaciones en recursos biológicos Alexander von Humboldt, Villa de Leyva, Boyacá, Colombia.

**ICN**Instituto de Ciencias Naturales, Universidad Nacional de Colombia, Bogotá D.C., Colombia.

**INPA** Instituto Nacional de Pesquisas da Amazônia, Manaus, Brazil

**MEFLG**Museo Entomológico Francisco Luis Gallego, Universidad Nacional de Colombia, Medellín, Colombia.

**MPEG**Museo Paraense Emilio Goeldi, Belem, Pará, Brazil.

**MUSENUV**Museo de Entomología de la Universidad del Valle, Valle del Cauca, Santiago de Cali, Colombia.

**MZSP** Museu de Zoologia da Universidade de São Paulo, São Paulo, Brazil

**NHMW**Naturhistorisches Museum, Wien, Austria.

**USNM**National Museum of Natural History, Washington, DC, USA.

## ﻿Results

### ﻿Taxonomic list of *Rhopalothrix* in Colombia

*Rhopalothrixamati* (Fiorentino, Tocora & Fernández, 2022), comb. nov.

*Rhopalothrixciliata* Mayr, 1870

*Rhopalothrixisthmica* (Weber, 1941)

*Rhopalothrixmandibularis* Guerrero & Grajales, sp. nov.

*Rhopalothrixmariaemirae* Tocora, Fiorentino & Fernández, sp. nov.

*Rhopalothrixweberi* Brown & Kempf, 1960

### ﻿Key to Colombian *Rhopalothrix* species based on workers

**Table:** 

1	Face with conspicuous squamiform setae (Fig. 3A)	**2**
–	Face lacking large squamiform setae (Fig. 6A) (*R.isthmica* clade)	**4**
2	Head elongate in full-face view, wider posterad than anterad. Lateral cephalic margin above antennal insertion straight and continuous, curved inwards posteriorly (Fig. 4A). Rounded occipital corner (Fig. 5A)	***R.ciliata* Mayr**
–	Head subquadrate in full-face view, almost as wide posterad as anterad. Lateral cephalic margin above the antennal insertion discontinuous, projecting outward over half of its length. Angled occipital corner (Figs 3A, 8A)	**3**
3	Mandible triangular, with curved external margin and straight masticatory margin. Masticatory margin of mandible with a row of teeth (Fig. 3C). Face with 12 specialized spatulate setae (6 in the anterior row, 6 in the posterior row, Fig. 3A)	***R.amati* (Fiorentino, Tocora & Fernández)**
–	Mandible elongated and arched, with the external and masticatory margins subparallel to each other (Figs 8A, 9). Masticatory margin of the mandible with only two teeth near the subapical tooth (Fig. 9). Face with single posterior row of 8 specialized spatulate setae (Figs 8A, 9)	***R.mariaemirae* sp. nov.**
4	In lateral view, mandible dorsally inclined in relation to head plane (Fig. 7B). Mandible elongated (MdL > 0.5) with five teeth on masticatory margin (Fig. 7A). Labrum about as long as broad (LabW/LabL < 100%), medial notch deep. Petiole with well-developed peduncle (Fig. 7A)	***R.mandibularis* sp. nov**.
–	In lateral view, mandible oriented in the same plane as the head (Figs 6B, 10B). Mandible short (MdL < 0.3), subtriangular (Figs 6A, 10A), and wide at base, with 2–3 teeth on masticatory margin. Labrum distinctly broader than long (LabW/LabL > 110%), medial notch shallow (Figs 6A, 10A). Petiole with short peduncle (Figs 6B, 10B)	**5**
5	Head broader than long, with slightly rounded cephalic lateral margins at the level of the crest on the face (Fig. 6A). Posterior cephalic margin strongly concave. Mandible with three teeth on masticatory margin, middle tooth largest (Fig. 6A). Subapical tooth longer than width of mandible at base, about twice as long as apical tooth	***R.isthmica* (Weber)**
–	Head as broad as it is long, with cephalic lateral margins projecting at an angle at the level of the crest on the face (Fig. 10A). Posterior cephalic margin slightly concave. Mandible with only two small teeth at the base of the masticatory margin (Fig. 10A). Subapical tooth shorter than width of mandible at base, only slightly longer than apical tooth	***R.weberi* Brown & Kempf**

### ﻿Species accounts

#### ﻿ Rhopalothrix amati

Taxon classificationAnimaliaHymenopteraFormicidae

(Fiorentino, Tocora & Fernández, 2022) comb. nov.

6F7A52B2-3D77-5073-B9E6-39071D650EBB

Figs 2 , 3

Eurhopalothrix amati Fiorentino, Tocora & Fernández, 2022: 3, figs 2, 3, 4 A, C. Holotype worker. IAvH-E-55017. Examined.

##### Worker measurements

**(*N* = 5).**MdL 0.16–0.2, MdbW 0.06–0.07, ClyL 0.12–0.15, ClyW 0.26–0.29, HL 0.38–0.42, HW 0.39–0.43, WL 0.4–0.46, PrnW 0.24–0.3, PetL 0.18–0.23, PpetL 0.09–0.11, PetW 0.13–0.15, PpetW 0.2–0.24, T4L 0.33–0.39, GL 0.42–0.48.

##### Geographic range.

Colombia.

##### Examined type material.

***Holotype*.** Colombia • 1 worker; Risaralda, Pereira, SFF El Otún Quimbaya, Vda. La Suiza, Plantación Urapán 7; 4.7321972°N, 75.578869°W; 1870 m a.s.l.; M.F. Reina & L.E. Franco legs.; sifted litter; IAvH-E-55017. ***Paratype*.** Colombia • 1 worker; Risaralda, Pereira, vda. La Suiza, Finca el Amparo de Niños; 4.7466278°N, 75.596939°W; 1810 m a.s.l.; 28–30 Nov. 2002; L.E. Franco leg.; secondary growth forest, ex sifted leaf litter; IAvH-55018.

##### Additional examined material.

Colombia • 1 worker; Antioquia, Támesis, vda. Alacena, Finca Villa Fátima; 5.2829167°N, 75.474139°W; 1940 m a.s.l.; 2 Oct. 2003; R. García leg.; IAvH-25326. • 3 workers; Caldas, Aranzazú, Vda. La Guaira, Finca Chambery; 5.7130556°N, 75.721833°W; 1900 m a.s.l.; 1–3 Jul. 2003; L.E. Franco & J. Cruz legs; ex sifted leaf litter, secondary growth forest; IAvH-55012.

**Figure 2.**
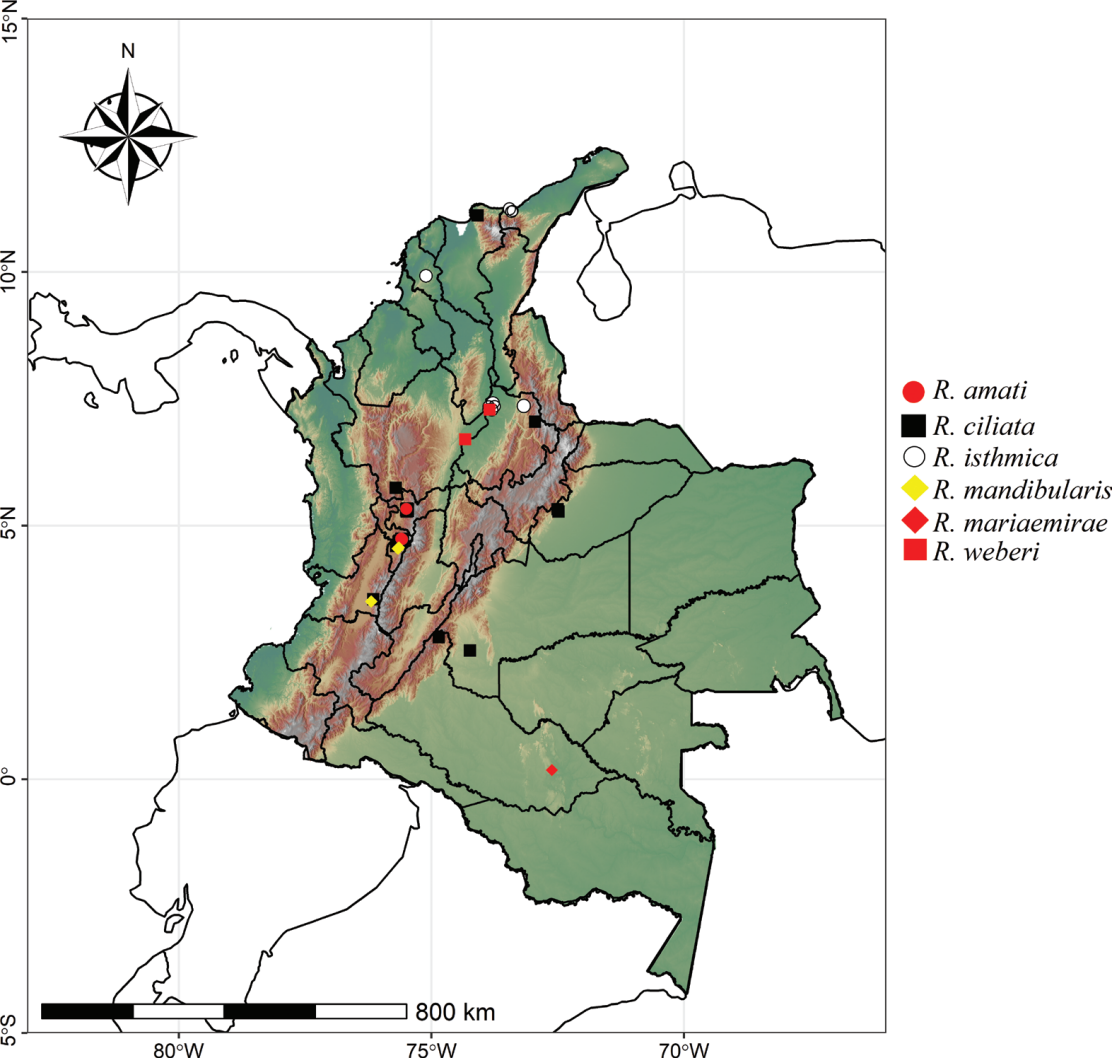
Distribution map of *Rhopalothrix* species in Colombia.

##### Comments.

**Figure 3.**
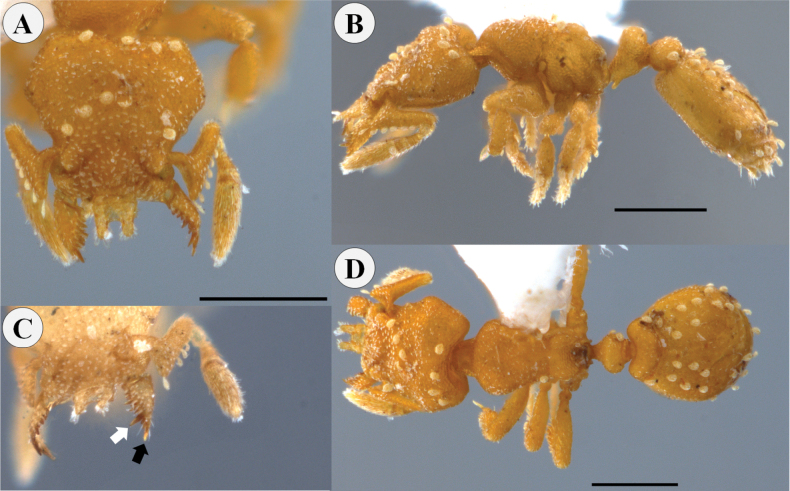
*Rhopalothrixamati* paratype worker (IAvH-55018) **A** full-face view **B** lateral view **C** dorsal view **D** portion of the head viewed obliquely showing the mandibles and the apical fork of the left mandible; the black arrow points to the subapical tooth while the white one points to the apical tooth of the apical fork of the mandible. Scale bars: 0.2 mm.

Holotype and paratype workers (IAvH-55017 and IAvH-55018) and three non-type specimens were analyzed and measured (HW 0.39–0.43) showing a mandibular dentition different from either of the two states described for *Eurhopalothrix* (Longino 2013). Fiorentino et al. (2022) indicate that the workers of this species have “masticatory margin with a single row of ~13 long needle shaped teeth…”, but this dentition does not match the simple row of 11 similar, low, triangular teeth mentioned for *Eurhopalothrix* by Longino (2013). Reanalyzing the mandible dentition of the workers, they present a row of between seven (IAvH55005 in AntWeb 2023) to ten teeth (holotype). The shape of the mandible of the workers of this species also does not match those of *Eurhopalothrix*, being more like the mandible of some undescribed *Rhopalothrix* (e.g., CASENT0639185*Rhopalothrix* jtl014 or CASENT0646264*Rhopalothrix* jtl023). It is possible that the triangular shape of the mandible of *Rhopalothrixamati* has generated the misclassification of this species in the genus *Eurhopalothrix*. All the workers studied, however, have the subapical tooth larger than the apical one (Fig. 3C), the latter being below the subapical tooth, a combination of traits present in *Rhopalothrix*. Based on all morphological evidence, we transfer this species to the genus *Rhopalothrix* generating the following new combination *Rhopalothrixamati* (Fiorentino, Tocora & Fernández, 2022).

#### ﻿ Rhopalothrix ciliata

Taxon classificationAnimaliaHymenopteraFormicidae

Mayr, 1870

F8F9B5F2-2B3B-5ADB-98F2-3462506B3FFD

Figs 2 , 4 , 5

##### Type material.

***Lectotype*.** Colombia • 1 worker; Santa Fe de Bogota; G. Mayr, leg.; AntWeb image examined, CASENT0915695; NHMW.

##### Worker measurements

**(*N* = 13).**MdL 0.3–0.36, MdbW 0.05–0.09, ClyL 0.15–0.22, ClyW 0.32–0.47, HL 0.43–0.81, HW 0.49–0.72, WL 0.49–0.8, PrnW 0.29–0.45, PetL 0.28–0.35, PpetL 0.1–0.16, PetW 0.14–0.21, PpetW 0.23–0.37, T4L 0.43–0.68, GL 0.52–0.86.

##### Geographic range.

Colombia, Ecuador, Venezuela. In Colombia, this species is known from Antioquia, Cundinamarca, Huila, Magdalena (Sierra Nevada de Santa Marta), Quindío and Valle del Cauca (Fernández and Serna 2019). New records in Colombia come from Caldas, Risaralda, and Santander.

##### Examined material.

Colombia • 4 workers; Antioquia, Támesis, Vda. LaVirgen Fca La Cumbre; 5.74531°N, 75.70542°W; 1610 m a.s.l.; 18 Aug. 2003; E. Patiño, leg.; winkler, low vegetation (stubble); IAvH 25286 to IAvH 25289. • 3 workers; Caldas, Aranzazu, Vda. Buenavista, Fca. La Palma; 5.27956°N, 75.49238°W; 2025 m a.s.l.; 29–31 Jul. 2003; L.E. Franco & J. Cruz legs.; winkler, living fence; IAvH 25010. • 1 worker; Caldas, Aranzazu, Vda. Chamberry, Fca. Las Garzas; 5.301939°N, 75.50144°W; 1940 m a.s.l.; 31 Jul.-4 Aug. 2003; L.E. Franco & J. Cruz legs.; winkler, mature forest fragment; IAvH 248793. • 1 worker; Caldas, Aranzazu, Vda. El Edén, Fca. El Gibarito; 5.29681°N, 74.8867°W; 1930 m a.s.l.; 5–7 Aug. 2003; L.E. Franco & J. Cruz legs.; winkler, riparian vegetation; IAvH 56368. • 2 workers; Caldas, Aranzazu, Vda. Guiaira, Fca. Villa Ofelia; 5.28549°N, 75.46419°W; 1965 m a.s.l.; 1–3 Aug. 2003; L.E. Franco & J. Cruz legs.; winkler, riparian vegetation; IAvH 54998. • 1 worker; Caldas, Aranzazu, Vda. La Guaira, Fca. Alto Bonito; 5.27883°N, 72.48461°W; 2056 m a.s.l.; 25–26 Jul. 2003; L.E. Franco & J. Cruz legs.; winkler; IAvH 56374. • 1 worker; Caldas, Aranzazu, Vda. La Pradera, Fca. Mina Manzanillo; 5.32169°N, 75.50144°W; 2080 m a.s.l.; 2–4 Aug. 2003; L.E. Franco & J. Cruz legs.; winkler, mature forest fragment; IAvH 55000. • 1 worker; Caldas, Aranzazu, Vda. San José, Fca. El Montier; 5.32694°N, 72.99028°W; 1960 m a.s.l.; 2–4 Jul. 2003; L.E. Franco & J. Cruz legs.; winkler, secondary forest fragment; IAvH 25012. • 1 worker; Caldas, Aranzazu, Vda. San José, Fca. Santa Teresa; 5.32475°N, 75.49786°W; 2005 m a.s.l.; 2–4 Aug. 2003; L.E. Franco & J. Cruz legs.; winkler; IAvH 56356. • 1 worker; Caquetá, PNN Picachos; 2.7975°N, 74.8549°W; 1775 m a.s.l.; Nov. 1997; F. Escobar leg.; ICN-MHN 080314. • 1 worker; Quindío, Armenia, Parque de la Vida; 4.5461398°N, 75.65933°W; 151 m a.s.l.; 8 Oct. 2020; A.F. Grajales-Andica & D.R. García-Cárdena legs.; winkler, bamboo forest; CBUMAG:ENT:35948.• 1 worker; Quindío, Circasia, Fca. Calamar; 5.9778°N, 75.7°W; 1450 m a.s.l.; 12 Oct. 1999; E. González leg.; winkler; IAvH 110900. • 1 worker; same data as for preceding; IAvH 80377. • 3 workers; Quindío, Filandia, Vda. Cruces, Fca. Agua Bonita; 4.68581°N, 75.62822°W; 1830 m a.s.l.; 20–22 Jul. 2002; E. Jiménez & L.E. Franco legs.; winkler, riparian vegetation; IAvH 56350; • 1 worker; Quindío, Filandia, Vda. Cruces, Fca. Agua Bonita; 4.68778°N, 75.62729°W; 1870 m a.s.l.; 21–23 Jul. 2002; E. Jiménez & L.E. Franco legs.; winkler, riparian vegetation; IAvH 56343. • 1 worker; Quindío, Filandia, Vda. Cruces, Fca Brasil; 4.68817°N, 75.64245°W; 1850 m a.s.l.; 24–26 Jul. 2002; E. Jiménez & L.E. Franco legs.; winkler, forest fragment; IAvH 56355. • 1 worker; Quindío, Filandia, Vda. Cruces, Fca. El Palacio; 4.69325°N, 75.63291°W; 1810 m a.s.l.; 18–20 Jul. 2002; E. Jiménez & L.E. Franco legs.; winkler, forest edge; IAvH 56358. • 1 worker; Quindío, Filandia, Vda. Cruces, Fca El Roble; 4.68239°N, 75.65247°W; 1990 m a.s.l.; 3–5 Jul. 2002; E. Jiménez & L.E. Franco legs.; winkler; IAvH 56365. • 3 workers; Quindío, Filandia, Vda. Cruces, Fca. La Cha; 4.70468°N, 75.62649°W; 1920 m a.s.l.; 28–30 Jul. 2002; E. Jiménez & L.E. Franco legs.; winkler, forest; IAvH 56359. • 1 worker; Quindío, Filandia, Vda. Cruces, Fca La Tunja; 4.68475°N, 75.65247°W; 2000 m a.s.l.; 17–19 Jul. 2002; E. Jiménez & L.E. Franco legs.; winkler, forest fragment; IAvH 56372. • 1 worker; Quindío, Filandia, Vda. Cruces, Fca Paraiso; 4.695°N, 75.62278°W; 1870 m a.s.l.; 4–6 Jun. 2002; E. Jiménez & M.F. Reina, legs.; winkler, forest; IAvH 25870. • 3 workers; Quindío, Filandia, Vda. Cruces, Fca Paraiso; 4.69767°N, 75.62582°W; 1910 m a.s.l.; 27–29 Jul. 2002; E. Jiménez & L.E. Franco legs.; winkler, forest; IAvH 56348. • 1 worker; same data as for preceding; IAvH-E-112817. • 1 worker; same data as for preceding; IAvH-E-112817. • 1 worker; Quindío, Filandia, Vda. Cruces, Fca Paraiso; 4.69278°N, 75.62009°W; 1910 m a.s.l.; 7–9 Jul. 2002; E. Jiménez & M.F. Reina, legs.; winkler, riparian vegetation; IAvH 56361. • 1 worker; same data as for preceding; IAvH-E-248916. • 1 worker; same data as for preceding; IAvH 56342. • 1 worker; Quindío, Filandia, Vda. Cruces, Fca Paraiso; 4.69302°N, 75.62009°W; 1910 m a.s.l.; 7–9 Jul. 2002; E. Jiménez & M.F. Reina, legs.; winkler, riparian vegetation; IAvH 56357. • 3 workers; same data as for preceding; IAvH 56362. • 1 worker; Quindío, Filandia, Vda. Cruces, Fca Paraiso; 4.69302°N, 75.62009°W; 1910 m a.s.l.; 12–14 Jul. 2002; E. Jiménez & L.E. Franco legs.; winkler, riparian vegetation; IAvH 56349. • 3 workers; Quindío, Filandia, Vda. Cruces, Fca Veracruz; 4.695°N, 75.60217°W; 28–30 Jul. 2002; 2010 m a.s.l.; E. Jiménez & L.E. Franco legs.; winkler, forest fragment; IAvH 56354. • 1 worker; Quindío, Filandia, Vda. Cruces, Fca Veracruz; 4.70317°N, 75.62945°W; 2010; 5–7 Jul. 2002; E. Jiménez & M.F. Reina, legs.; winkler, plantation; IAvH 56351. • 3 workers; Risaralda, Pereira, Vda. La Aurora, Fca. Los Balcones; 5.32714°N, 75.46688°W; 1957 m a.s.l.; 30 Jul-1 Aug. 2003; L.E. Franco & E. Londoño legs.; winkler, secondary forest fragment; IAvH 25007. • 1 worker; Risaralda, Pereira, Vda. La Suiza Fca. Cartón Colombia; 4.72544°N, 75.60016°W; 2100 m a.s.l.; 21–23 Nov. 2003; M.F. Reina & L.E. Franco legs.; winkler, Eucalyptus plantation; IAvH 25001. • 2 workers; Risaralda, Pereira, Vda. La Suiza, Fca. El Amparo de Niños; 4.7455°N, 75.59672°W; 1840 m a.s.l.; 28–30 Nov. 2003; L.E. Franco & E. Londoño legs.; winkler, mature forest; IAvH 24995. • 3 workers; Risaralda, Pereira, Vda. La Suiza, Fca. Lisdrand; 4.74293°N, 75.58028°W; 1930 m a.s.l.; 4–6 Dec. 2003; L.E. Franco & E. Londoño legs.; winkler, mature forest; IAvH 56360. • 1 worker; Risaralda, Pereira, Vda. La Suiza, Fca. Tesorito; 4.72236°N, 75.560108°W; 2050 m a.s.l.; 27–29 Nov. 2003; L.E. Franco & E. Londoño legs.; winkler, mature forest; IAvH 56366. • 1 worker; Risaralda, Pereira, Vda. La Suiza, SFF Otún Quimbaya; 4.71962°N, 75.58028°W; 1910 m a.s.l.; 26 Feb. 2003; L.E. Franco & E. Londoño legs.; winkler, urapán plantation; IAvH 56371. • 2 workers; Risaralda, Pereira, Vda. La Suiza, SFF Otún Quimbaya; 4.71962°N, 75.580423°W; 1910 m a.s.l.; 11–13 Jan. 2003; L.E. Franco & E. Londoño legs.; winkler, urapán plantation; IAvH 24999. • 1 worker; Risaralda, Salamina, Vda. En Medio de Rio, Fca. Villa Belmira; 5.33563°N, 75.48236°W; 1740 m a.s.l.; 29–31 Jul. 2003; L.E. Franco & J. Cruz, legs.; winkler, shade-grown coffee; IAvH 25003. • 1 worker; Santander, Piedecuesta, Cgto. Sevilla Vda. Cristales, reserva experimental demostrativa El Rasgón; 7.05°N, 72.95°W; 2150 m a.s.l.; 21–23 Sep. 2004; I. Quintero & E. González legs.; winkler, high Andean forest; IAvH 71848. • 1 queen; Valle del Cauca, vda. La Quisiquina, Finca Casa Blanca; 3.55°N, 76.15°W; 1914 m a.s.l.; Aug. 2006; Grupo hormigas UV., legs.; ex sifted leaf litter, forest fragment; MUSENUV HOR 006. • 1 worker; same data as for preceding; MUSENUV HOR 007. • 1 worker; same data as for preceding; IAvH-E-248789. • 1 worker; same data as for preceding; IAvH-E-248886.

**Figure 4.**
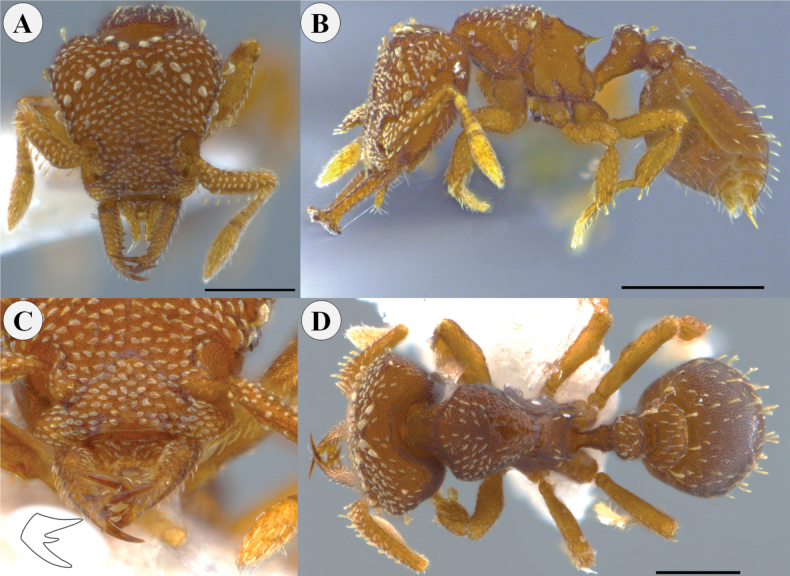
*Rhopalothrixciliata* worker (CBUMAG:ENT:35948) **A** full-face view **B** lateral view **C** mandible distinguishing the teeth of the apical fork; drawing inserted showing the arrangement of the teeth of the apical fork of the mandible **D** dorsal view. Scale bars: 0.3 mm (**A, D**); 0.5 mm (**B**).

##### Natural history.

In Colombia, this species is known from forests at altitudes above 1500 m, with populations in the Sierra Nevada de Santa Marta and in regions of the central and western cordilleras. It is a very abundant species in modified environments and in agroecosystems such as coffee crops that include native trees.

##### Comments.

**Figure 5.**
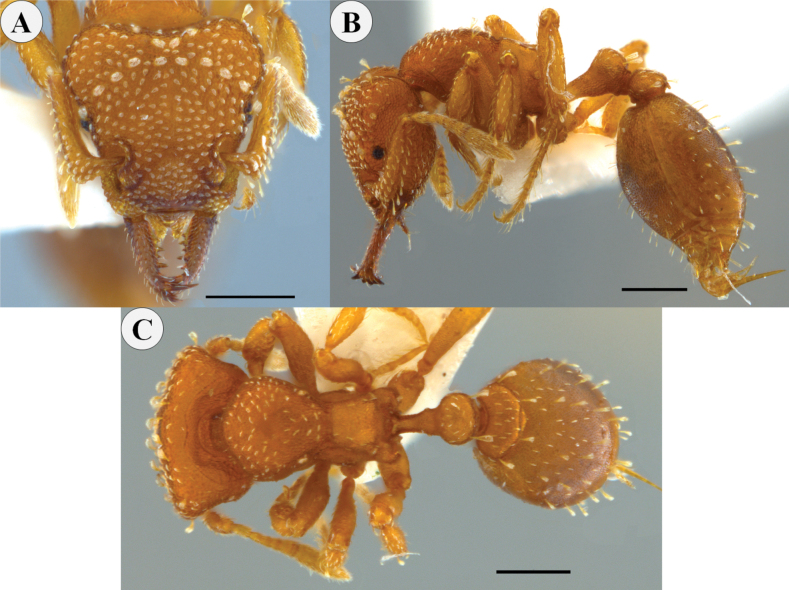
*Rhopalothrixciliata* worker (ICN80314) **A** full-face view **B** lateral view **C** dorsal view. Scale bars: 0.2 mm.

The specimen from Caquetá (ICN-MHN 080314) is the largest worker (HW 0.72, WL = 0.8; Fig. 5) known so far compared to the other workers (HW 0.49–0.63, *N* = 12) studied here and the lectotype (HW 0.66, WL = 0.75; from Brown and Kempf 1960). A worker (IAvH 110900) from Quindío is the smallest (HW 0.49).

#### ﻿ Rhopalothrix isthmica

Taxon classificationAnimaliaHymenopteraFormicidae

(Weber, 1941)

AC984301-36BE-5980-B931-89F3D31BC46F

Figs 2 , 6

##### Worker measurements

**(*N* = 8).**MdL 0.28–0.3, MdbW 0.08–0.09, ClyL 0.14–0.19, ClyW 0.4–0.43, HL 0.51–0.53, HW 0.54–0.59, WL 0.51–0.56, PrnW 0.29–0.32, PetL 0.23–0.3, PpetL 0.12–0.15, PetW 0.16–0.17, PpetW 0.28–0.31, T4L 0.49–0.55, GL 0.55–0.58.

##### Geographic range.

Colombia, Honduras, Guatemala, Panama. In Colombia, this species is known from Antioquia, Bolívar, Santander and Sucre.

##### Examined material.

Colombia • 1 worker; Antioquia, Amalfi, cañon del Porce, La Cancana; 6.76667°N, 74.91667°W; 1000 m a.s.l.; 30 Jul. 1997; F. Serna leg.; ex sifted leaf litter mini-Winkler, low vegetation (stubble); MEFLG 11112. • 1 worker; Bolívar, SFF Los Colorados, La Yaya; 9.92611°N, 75.10583°W; 280 m a.s.l.; 3–5 Jul. 2001; ex sifted leaf litter, dry forest; IAvH-E-263435. • 3 workers; La Guajira, Dibulla, Bello Horizonte, río Cañas; 11.25687°N, 73.44852°W; 6 m a.s.l.; 12 Oct. 2015; ex sifted leaf litter, dry forest; IAvH-E-172164. • 3 workers; La Guajira, Dibulla, Alto San Jorge, río Cañas; 11.218°N, 73.428°W; 73 m a.s.l; 12 Oct. 2015; ex sifted leaf litter, dry forest; IAvH-E-172162, IAvH-E-172163, IAvH-E-172165. • 1 worker; Santander, Rionegro, Vereda Galapagos, Km 32 vía al mar, C.I. La Zuiza; 7.370278°N, 73.17762°W, 537 m a.s.l.; 2020; J.M. Montes leg.; CTNI 8304. • 2 workers; Santander, Puerto Wilches, Platero; 7.3483°N, 73.8960°W; 28 m a.s.l.; 10–15 Nov. 2021; ex sifted leaf litter Winkler No. 4; L. Perez leg.; CBUMAG:ENT:35949. • 1 worker; Santander: Puerto Wilches, Vereda Puente Sogamoso; 7.30537°N, 73.82779°W; 87 m a.s.l.; 22 Jul. 2022; L. Velázquez leg.; IAvH-E-226990. • 14 workers; Santander, Puerto Wilches, Vereda Centro, 7.32972°N, 73.84256°W; 88 m a.s.l.; 8 Jul. 2022; L. Arcila leg; ex sifted leaf litter, riparian forest; IAvH-E-226992, IAvH-E-226993, IAvH-E-226994, IAvH-E-226995, IAvH-E-226996, IAvH-E-232292, IAvH-E-233744, IAvH-E-238973, IAvH-E-238974, IAvH-E-238975, IAvH-E-238976, IAvH-E-243661, IAvH-E-243688. • 1 worker; Santander: Puerto Wilches, Vereda San Claver; 7.34831°N, 73.76817°W; 93 m a.s.l.; 8 Jul. 2022; C. Quevedo-Vega leg.; ex sifted leaf litter, riparian forest; IAvH-E-226997. • 1 worker; Santander: Puerto Wilches, Vereda San Claver; 7.34792°N, 73.76817°W; 77 m a.sl.; 8 Jul. 2022; C. Quevedo-Vega leg.; ex sifted leaf litter, riparian forest; IAvH-E-226998.

##### Natural history.

*Rhopalothrixisthmica* workers inhabit dry forest in northern Colombia and both in open grassland and riparian forest in eastern Colombia. In the latter it is a relatively abundant species, being found in 8 of 20 MiniWinkler litter samples. *Rhopalothrixisthmica* populations have an elevational distribution from near sea level to 1000 m.

##### Comments.

The morphology of the workers matches the diagnostic characters of *R.isthmica*, including HW 0.57–0.61 (*N* = 6) recorded by Longino and Boudinot (2013). In the workers of populations from La Guajira (northern Colombia), the portion of the lamella ventral to the propodeal tooth (infradental lamella) is relatively straighter on the outer margin than described by Longino and Boudinot (2013).

**Figure 6.**
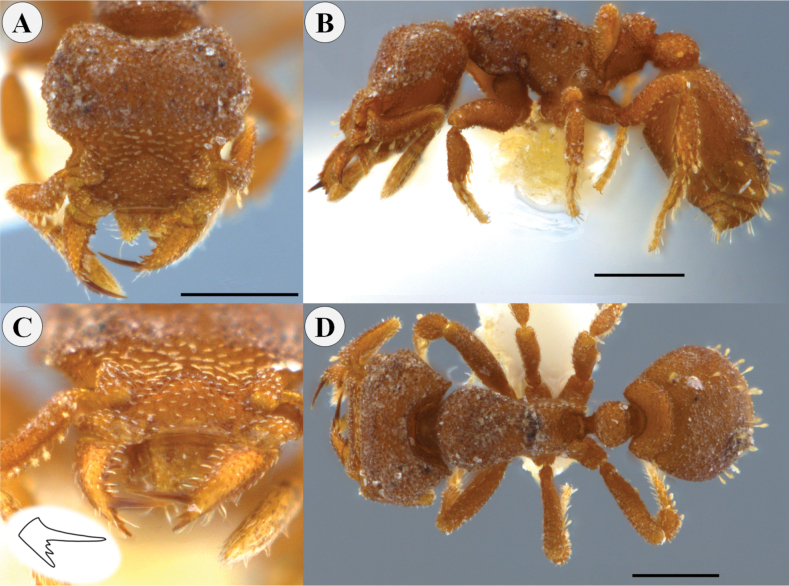
*Rhopalothrixisthmica* worker (IAvH-E-172166) **A** full-face view **B** lateral view **C** mandible distinguishing the teeth of the apical fork; drawing inserted showing the arrangement of the teeth of the apical fork of the mandible **D** dorsal view. Scale bars: 0.3 mm.

#### ﻿ Rhopalothrix mandibularis

Taxon classificationAnimaliaHymenopteraFormicidae

Guerrero & Grajales sp. nov.

1AB4C61B-C603-5619-BD1B-FBB433E65387

https://zoobank.org/F063C899-9938-4D48-B1B1-BAA604287043

Figs 2 , 7

##### Type material.

***Holotype*.** Colombia • 1 worker; Quindío, Armenia, Sena; 4.56931°N, 75.64347°W; 1565 m a.s.l.; 18 Feb. 2020; A.F. Grajales-Andica & D.R. García-Cárdenas legs.; ex sifted leaf litter, gallery forest; CBUMAG:ENT:35947. ***Paratypes*** (*N* = 4). Colombia • 1 worker; same data as for holotype; CIUQ-025287. • 1 worker; Quindío, Armenia, Parque de la Vida; 4.54614°N, 75.65933°W; 1515 m a.s.l.; 8 Oct. 2020; A.F. Grajales-Andica & D.R. García-Cárdenas legs.; ex sifted leaf litter, gallery forest; CIUQ-025288. • 1 worker; Quindío, Armenia, Yulima; 4.5515°N, 75.671°W; 1485 m a.s.l.; 8 Feb. 2020; A.F. Grajales-Andica & D.R. García-Cárdenas legs.; ex sifted leaf litter, gallery forest; CIUQ-025289. • 1 worker; Valle del Cauca, Vda. El Tenjo, Finca La Alejandría; 3.51667°N, 76.16667°W; 1703 m a.s.l.; Aug. 2006; Grupos Hormigas U. V. legs.; ex sifted leaf litter; MUSENUV HOR 008.

##### Holotype worker measurements.

MdL 0.48, MdbW 0.08, ClyL 0.2, ClyW 0.44, HL 0.67, HW 0.76, WL 0.65, PrnW 0.43, PetL 0.36, PpetL 0.13, PetW 0.19, PpetW 0.31, T4L 0.6, GL 0.68.

##### Paratype workers measurements

**(*N* = 3).**MdL 0.48–0.52, MdbW 0.08–0.09, ClyL 0.19–0.21, ClyW 0.44–0.47, HL 0.65–0.69, HW 0.76–0.78, WL 0.65–0.69, PrnW 0.43–0.45, PetL 0.36–0.38, PpetL 0.13–0.17, PetW 0.19–0.22, PpetW 0.3–0.31, T4L 0.6–0.73, GL 0.67–0.73.

##### Geographic range.

Colombia.

##### Diagnosis.

Mandible elongated, much longer (MdL > 0.48) than those of other species in the *isthmica* clade, mandibles with outer and masticatory margins subparallel to each other and curving inward at tip; labrum with two slender subrectangular lobes, notch deep; propodeal tooth large, acute, right angled to declivitous face of propodeum, infradental lamella poorly developed, forming a thin rim.

##### Description.

**Figure 7.**
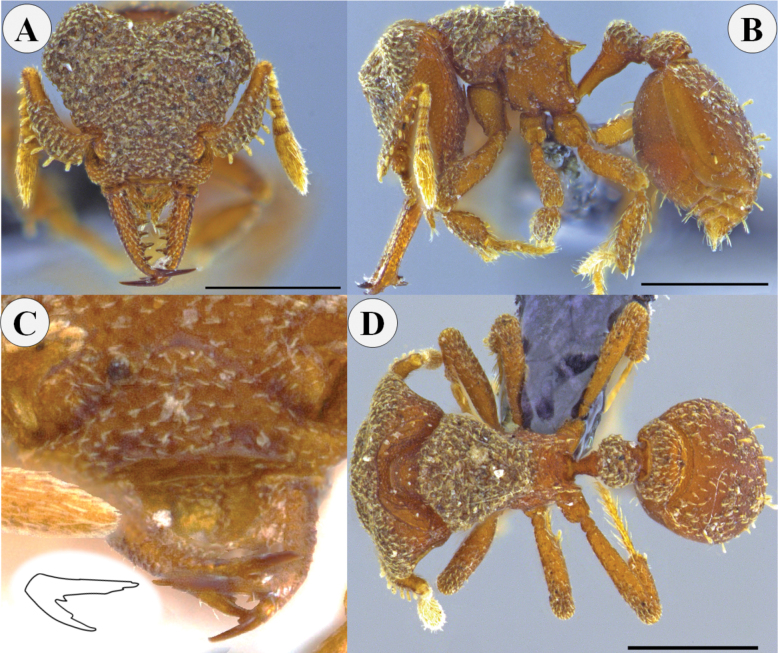
*Rhopalothrixmandibularis* sp. nov. Holotype worker (CBUMAG:ENT:35947) **A** full-face view **B** lateral view **C** mandible distinguishing the teeth of the apical fork; drawing inserted showing the arrangement of the teeth of the apical fork of the mandible **D** dorsal view. Scale bars: 0.5 mm.

**Worker.** Head in full-face view broader than long, diamond-shaped, with straight cephalic lateral margins strongly diverging posteriorly, extending below the level of the dorsal crest of the head, at the level of the latter a rounded widening that continues on lateral margins converging towards the rounded posterolateral cephalic corners; wide and concave posterior cephalic margin; front visibly protruding in dorsal view, with an arched transverse carina (= crest), and depression impressed behind the crest. In lateral view, mandible dorsally inclined in relation to head plane (Fig. 7B); mandible with four to five teeth on masticatory margin as follow: three large equidistant teeth located medially on masticatory margin, basalmost (first) large tooth with a small tooth (sometimes undeveloped) above its base, a middle tooth almost half as long as the previous one, third tooth as long as first, a small fourth tooth as long as 1/3 of first; subapical tooth about twice as long as apical tooth, with denticles at base of both subapical and apical tooth. Trapezoidal labrum as long as broad, with slightly concave sides, subparallel anteriorly, and straight-sided base, labrum with two long blunt subrectangular lobes, with parallel inner faces and deep notch between, length of lobe equal to about 1 /3 of the distance from the base of the notch to the transverse carina at the base of the labrum, Clypeus almost twice as wide as long, with anteroclypeal lobes projecting anterad. Scape just reaching maximum width of head; pedicel and second flagellomere conical towards the base as long as wide, third and fourth flagellomere rectangular wider than long, fifth flagellomere subsquare, last flagellomere finger-shaped tapering apically, as long as the previous four funiculus.

In lateral view, pronotum and mesonotum at the same level, divided by arcuate promesonotal groove and metanotal groove moderately impressed; propodeal dorsum sloping in lateral view. In dorsal view, pronotum with slightly concave anterior margins, narrowing anteriorly to form a distinguishable neck, pronotum with rounded corners at maximum width; in dorsal view, mesonotum wider than long, narrowing posteriorly. Petiole with well-developed peduncle; in lateral view, with straight dorsal face and posteriorly convex ventral face, the latter with a small subpetiolar process projecting anterad as a blunt tooth; petiolar scale rounded in lateral view; postpetiole twice as wide as long (Fig. 7C).

Short decumbent hairs on surface of head and mesosoma; dorsum of clypeal plate and above posteroclypeal margin with small squamiform hairs. External margin of scape with about 7–9 squamiform hairs similar in size; apex of scape with erect hairs shorter than squamiform ones; funicles with simple subdecumbent hairs. Legs with coxa and femur with few short decumbent hairs; tibia with abundant long, thick semi-erect hairs, and a pair of long flattened hairs located apically on the external surface of each one. About 4–8 squamiform setae on posterior half of first gastral tergite, unspecialized curved hairs scattered over the disc of the first gastral tergite.

Head, mesosoma, petiole and postpetiole shagreened, legs shiny with granular surface, except all tibiae with smooth surface; surface of first gastral tergite finely shagreened. Color reddish brown to ferruginous brown, with yellowish brown distal antennal flagellomeres.

##### Natural history.

This species inhabits humid forests between 1400 and 1700 m above sea level. The holotype and several paratypes were collected in fragments of humid gallery forest and Guadua (bamboo) forest in the city of Armenia. All known specimens are from Winkler samples of sifted leaf litter.

##### Etymology.

The name refers to the long mandibles of the worker, a trait not found in any other species in the *isthmica* clade.

##### Comments.

This species is placed in the diverse *isthmica* clade because it shares the two synapomorphies proposed by Longino and Boudinot (2013). This new species, however, has been widely confused in some Colombian collections with *R.ciliata* due to its similarity in mandibular shape, the shape of the two lobes of the labrum and the depth of the sinus. *Rhopalothrixmandibularis* can be differentiated from *R.ciliata* by the absence of squamiform setae on the rostrum, the latter with specialized setae and a flattened surface on the rostrum. Also, mandibles are longer and thinner along their length in *R.mandibularis* while in *R.ciliata* they are short and broad; in *R.ciliata* the tip of the labral lobes can reach up to half the length of the mandible, but in *R.mandibularis* the anterior margin of the labrum barely reaches the first tooth of the mandible.

Habitus of the worker of *Rhopalothrixmandibularis* is similar to that of *R.stannardi* Brown & Kempf, 1960, but the mandible length is remarkably different between the two, as well as the mandible dentition; *R.mandibularis* has three teeth located towards the middle of the masticatory margin (the most basal tooth is far from the base), while in *R.stannardi* the three teeth are equidistant, with the most basal tooth starting at the base of the masticatory margin. Another different feature is the infradental lamella, which is very poorly developed in *R.mandibularis*, while in *R.stannardi* the lamella is broad and descends almost perpendicularly from the tooth.

#### ﻿ Rhopalothrix mariaemirae

Taxon classificationAnimaliaHymenopteraFormicidae

Tocora, Fiorentino & Fernández sp. nov.

6415A869-F84D-5B98-985E-1265A625709D

https://zoobank.org/5EB90D5A-42C0-4D0F-815C-EDB577DD2782

Figs 1 , 8 , 9

##### Type material.

***Holotype*.** Colombia • 1 worker; Guaviare, Solano, PNN Serranía de Chiribiquete; 0.18189°N 72.61589°W; 250 m a.s.l.; 30 Nov. 2000; F. Acevedo leg.; ICNC: 099809. ***Paratypes*** (*N* = 7). • 1 worker; same data as holotype; CBUMAG:ENT:35950. BRAZIL • 1 worker; Amazonas, Manaus, 2.40262°S, 59.86655°W; 12 Aug. 2016; B. Boudinot, I. Fernandes I & J. Chaul; winkler; ANTWEB1038216; INPA. • 1 worker; same data as for preceding; UFV-LABECOL-001942; MZSP. • 1 worker; same data as for preceding; UFV-LABECOL-001945; MPEG. • 1 worker; same data as for preceding; UFV-LABECOL-001953; CELC. • 1 worker; same data as for preceding; UFV-LABECOL-007266; JTLC. • 1 worker; Amazonas, Manaus, 2.40372°S, 59.86573°W; 12 Aug. 2016; B. Boudinot, I. Fernandes I & J. Chaul; winkler; UFV-LABECOL-001944; DZUP.

##### Other examined material.

Colombia • 1 worker; Amazonas, Parque Nacional Natural Amacayacu; 3.81028°S, 70.2662°W; 88 m a.s.l.; 07 Oct. 2007; J. Sosa-Calvo & J. Rodriguez legs.; winkler, leaf litter, forest; USNMENT01127995; USNMENT01127995; USNM. Brazil • 1 worker; Amazonas, Manaus; 2.93333°S, 59.95°W; 6 Oct. 2006; J.L.P. Souza & J.S. Araújo legs.; ANTWEB1038211; INPA. • 1 worker; same data as for preceding; J.L.P. Souza & P.Y. Oliveira legs.; ANTWEB1038212; INPA. • 1 worker; Amazonas; 2.56669°S, 60.09999°W; 9 Sep. 1990; M.O. de A Ribeiro leg.; ANTWEB1038213; INPA. • 1 worker; Rondônia: Jaci Novo; 22 Oct. 2013; ANTWEB1038214; INPA. • 1 worker; Pará, Melgaço, Estação Científica Ferreira Penna; 1.71668°S, 51.41668°W; J.L.P. Souza & C. Moura; 26 Oct. 2003; ANTWEB1038215; INPA. • 1 worker; Rondônia, Porto Velho, área Mutum (M5P2); 9.591389°S, 65.04917°W; 17–27 Jul. 2013; G.R. Mazão & R.S. Probst legs.; CPDC. • 1 worker; Pará, Marituba; 1.36667°S, 48.33333°W; 20 m a.s.l.; 22 Oct. 2004; J.R.M. Santos leg.; winkler, mata; CPDC. GUYANA • 1 worker; Rupununi, nr. Kamoa River, nr Kamoa R. Camp; 1.55077°N, 58.83832°W; 535 m a.s.l.; 24 Oct. 2006; R. Williams & P. Suse legs.; winkler, leaf litter; USNMENT01127994; USNM.

##### Geographic range.

Colombia, Guyana, Brazil.

##### Holotype worker measurements.

MdL 0.3, MdbW 0.06, ClyL 0.16, ClyW 0.43, HL 0.48, HW 0.55, WL 0.52, PrnW 0.35, PetL 0.3, PpetL 0.12, PetW 0.19, PpetW 0.28, T4L 0.42, GL 0.56.

##### Paratype workers measurements

**(*N* = 7).**MdL 0.25–0.33, MdbW 0.06–0.08, ClyL 0.12–0.17, ClyW 0.38–0.44, HL0.39–0.49, HW 0.48–0.55, WL 0.43–0.52, PrnW 0.29–0.35, PetL 0.21–0.30, PpetL 0.09–0.13, PetW 0.16–0.19, PpetW 0.24–0.29, T4L 0.35–0.42, GL 0.42–0.56.

##### Diagnosis.

Masticatory margin of mandible with two small teeth near the base of the subapical tooth; labrum rounded, about as long as broad, with two poorly produced, bluntly, rounded anterior lobes; promesonotal and metanotal groove continuously concave; larger specialized hairs on face are shaped like inverted bowls of broad flat spoons lying close to and paralleling the integumental surface, in perpendicular view they look like eight large, rounded white scales on head.

##### Description.

**Worker.** Head in full-face view wider than long, with cephalic lateral margins subparallel to each other, profile interrupted by a deeply impressed notch at the level of the antennal insertions and a triangular notch shallower than the previous one below the level of the diadem of circular/squamiform hairs, at the level of the latter, widened profile projecting rounded angulations continuing on slightly convergent lateral margins towards the angled posterolateral cephalic corners; wide and strongly concave posterior cephalic margin. In lateral view, mandible in the same plane of the head; subapical tooth with prominent recurved acute tooth, directed posteriorly, subapical tooth shorter than width of mandible at base, about twice as long as apical tooth. In full-face view, anterior margin of labrum with shallow median notch, posteromedial portion of labrum translucid. Clypeus about 2.5 times wider than long, with rounded anteroclypeal lobes projecting anterad. Scape just beyond the most posterior notch of the lateral cephalic margin; Pedicel subsquare, second to fourth flagellomere conical towards the base, fifth flagellomere rectangular longer than wide, last flagellomere finger-shaped tapering towards the apex, almost as long as almost as long as the five flagellomeres combined.

**Figure 8.**
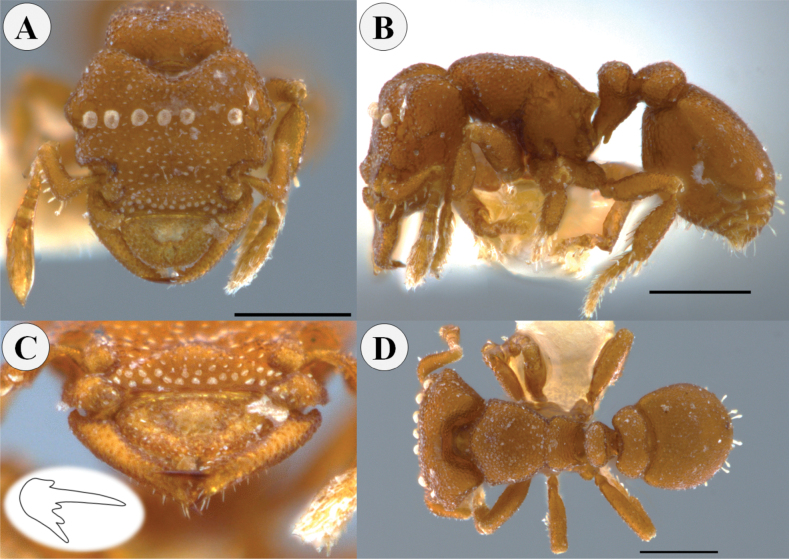
*Rhopalothrixmariaemirae* sp. nov. paratype worker (CBUMAG:ENT:35950) **A** full-face view **B** lateral view **C** mandible distinguishing the teeth of the apical fork; drawing inserted showing the arrangement of the teeth of the apical fork of the mandible **D** dorsal view. Scale bars: 0.3 mm.

In lateral view, promesonotum convex continuing with the profile of the dorsum of the propodeum, promesonotal depression and metanotal groove slightly impressed; propodeal dorsum falling on a slight slope in lateral view; propodeal tooth developed, distinctly in top half of declivitous face of propodeum in lateral view; infradental lamella very narrow. In dorsal view, pronotum with straight lateral and convex anterior margin, pronotum with angled corners at their maximum width that continue towards slightly convex lateral margins; mesonotum trapezoid-shaped, wider than long, narrowing posteriorly. Petiole with poor-developed peduncle; in lateral view, with the dorsal face short, strongly inclined to connect with the anterior face of the rounded petiole scale, ventral surface straight with a small elongated subpetiolar process projecting anterad; in dorsal view, postpetiole 1.5 times wider than width of petiole (Fig. 8C).

Head with short decumbent squamiform hairs, notably dispersed and arranged transversely; anterodorsal portion of clypeus with small squamiform hairs broadened apicad. External margin of the scape with about 6–7 squamiform hairs similar in size; apex of scape with few shorter and thicker erect hairs, widely scattered; flagellomeres with simple subdecumbent hairs. Coxa and femur with few very short hairs; tibiae with long, thick semi-erect squamiform hairs on the inner surface, external face of the tibiae devoid of erect hairs, with only a few long, flattened hairs located apically. First gastral tergite largely devoid of setae, with 2–3 squamiform setae at posterolateral margins.

**Figure 9.**
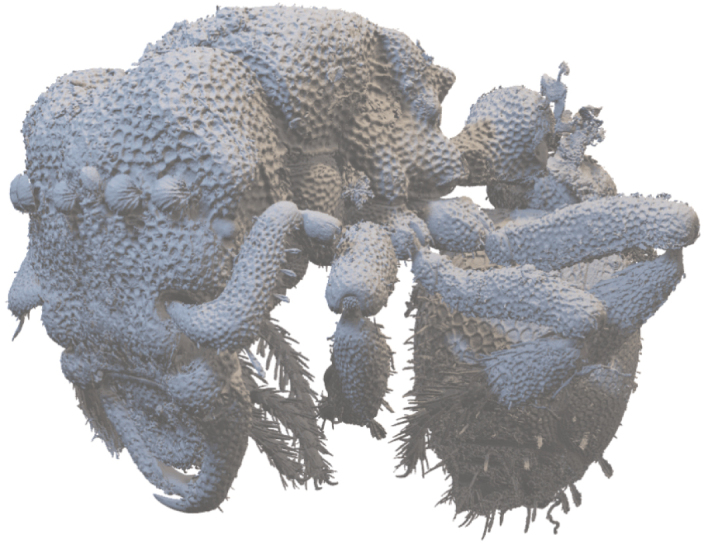
Volume render of *Rhopalothrixmariaemirae* sp. nov. worker.

Head, mesosoma, petiole and postpetiole shagreened, with the surface strongly areolate (Fig. 9); surface of first gastral tergite areolate. Color ocher to orange, concolorous.

**Queen and male.** Unknown.

##### Etymology.

This species is named after María Emir Sánchez (1953–2023), as a tribute to María C. Tocora’s beloved and inspiring *abuela*, who recently passed away.

##### Comments.

This species is easily recognized by the anterior labral convexity condition, the two small blunt lobes of the labrum, and the large squamate hairs, 8 in total, on the frons like those of *R.diadema* (Longino and Boudinot 2013).

The workers of *Rhopalothrix* jtl021 (ANTWEB1038216, UFV-LABECOL-001953, and USNMENT01127994) match to *R.mariaemirae*. Those specimens coincide in the strongly convex labrum, distal margin of labrum slightly notched, and the two poorly-developed lobes. In *Rhopalothrix* jtl021 the posteromedial portion of labrum is translucid. Also, all specimens share eight strongly convex rounded scales located below the maximum width of the head.

#### ﻿ Rhopalothrix weberi

Taxon classificationAnimaliaHymenopteraFormicidae

Brown & Kempf, 1960

88DFAFF5-F16F-51D8-8080-92FE5DCA333B

Figs 2 , 10

##### Worker measurements

**(*N* = 1).**MdL 0.16, MdbW 0.07, ClyL 0.12, ClyW 0.25, HL 0.36, HW 0.37, WL 0.38, PrnW 0.24, PetL 0.17, PpetL 0.09, PetW 0.14, PpetW 0.2, T4L 0.33, GL 0.41.

##### Geographic range.

Mexico, Guatemala, Honduras, Nicaragua, Costa Rica, Cuba, Colombia, Guyana, Suriname.

##### Examined material.

Colombia • 1 worker; Santander, Puerto Wilches, Vereda Centro; 7.32972°N, 73.84256°W; 87 m a.s.l.; 13 Jul. 2021; G. Mercado leg.; ex sifted leaf litter riparian forest; IAvH-E-233235.

##### Natural history.

The only specimen studied here was extracted from the low-density litter of a riparian forest with shrubby vegetation.

##### Comments.

This species is recorded by Achury and Suarez (2018) from the Colombian inter-Andean valley, but we were not able to study those specimens to corroborate the identity. Ants recently collected in Puerto Wilches (Santander) in eastern Colombia included one specimen that matches the taxonomic definition of *R.weberi*, thus corroborating the presence of this species in Colombia.

**Figure 10.**
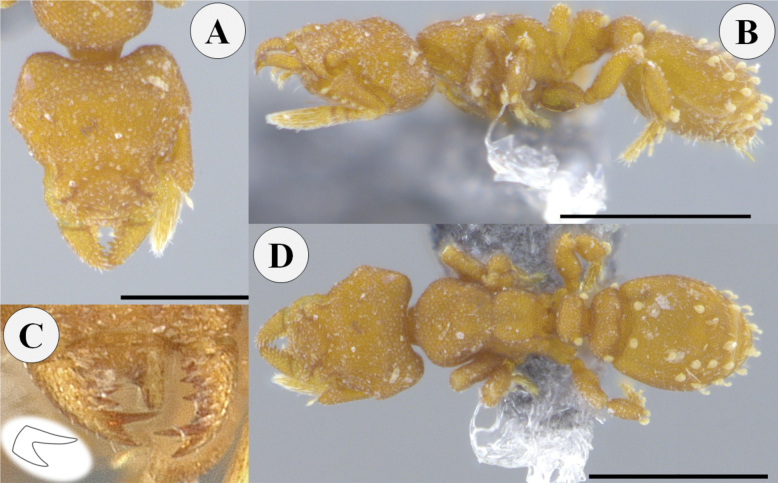
*Rhopalothrixweberi* worker (IAvH-E-233235). **A** full-face view **B** lateral view **C** mandible distinguishing the teeth of the apical fork; drawing inserted showing the arrangement of the teeth of the apical fork of the mandible **D** dorsal view. Scale bars: 0.3 mm.

### ﻿General comments

The ant genus *Rhopalothrix* is reviewed for the first time for Colombia. Previously only three species were known: *R.ciliata*, *R.isthmica* and *R.weberi* (Janicki et al. 2016); the last with an uncertain record for Antioquia (Achury and Suarez 2018). Our study increases the number of species to six, with the description of two new species, *R.mandibularis* and *R.mariaemirae*.

*Rhopalotrhixciliata* and *R.isthmica* are the most widely distributed species in Colombia, the first species with populations mainly in the Andean region and the Sierra Nevada de Santa Marta (northern Colombia), while *R.isthmica* has populations in dry forests of the Colombian Caribbean, in cleared open areas and in remnants of riparian forest in Santander in the valley of the eastern Cordillera of Colombia. Interestingly, *Rhopalotrhixciliata* overlaps its distribution with both *R.amati* and *R.mandibularis* in the coffee-producing region of central Colombia and in Valle del Cauca; in the latter, however, a disjunct altitudinal distribution is evident, as *R.ciliata* can be found above 2000 m while *R.mandibularis* is at 1700 m. Another example of sympatric distribution is recorded for *R.isthmica* and *R.weberi* in riparian forests in northeastern Colombia, where both species were found coexisting in leaf litter.

## Supplementary Material

XML Treatment for Rhopalothrix amati

XML Treatment for Rhopalothrix ciliata

XML Treatment for Rhopalothrix isthmica

XML Treatment for Rhopalothrix mandibularis

XML Treatment for Rhopalothrix mariaemirae

XML Treatment for Rhopalothrix weberi

## ﻿Additional information

### Author ORCIDs

Roberto J. Guerrero https://orcid.org/0000-0003-3244-2754

Andrés F. Grajales-Andica https://orcid.org/0000-0001-9166-0562

Fernando Fernández https://orcid.org/0000-0002-6862-3592

María C. Tocora https://orcid.org/0000-0002-5304-4010

Gianpiero Fiorentino https://orcid.org/0000-0001-6948-5032

Delly R. García https://orcid.org/0000-0002-4821-6754

### Data availability

All of the data that support the findings of this study are available in the main text or Supplementary Information.
